# The Pathology of Suppuration in the Nasal Accessory Sinuses and Its Relationship to Nasal Polypus

**Published:** 1899-08-05

**Authors:** Charles H. McIlraith

**Affiliations:** Senior Clinical Assistant (late Resident Medical Officer), the Throat Hospital, Golden Square, W.; late Aural Assistant, the London Hospital


### Hospital Clinics and Medical Progress.

### THE PATHOLOGY OF SUPPURATION IN THE NASAL ACCESSORY SINUSES AND ITS RELATIONSHIP TO NASAL POLYPUS.

By Charles H. McIlraith, M.A., M.D., &c. (Glasg.), Senior Clinical Assistant (late Resident Medical Officer), the Throat Hospital, Golden Square, W.; late Aural Assistant, the London Hospital.

(*Continued from page* 294.)

Now the maxillary antrum and the frontal and sphenoidal sinuses are single cavities, opening by comparatively small openings into the nasal passages. If the inflammatory process attacks the mucous membrane lining them their normal openings tend to be blocked up by the swollen membrane, and the discharge does not obtain a free exit. The swelling, however, is temporary in the acute disease, and consequently, after some days of discomfort to the patient, subsides, and there is a copious discharge of pus at once relieving all symptoms.

Unfortunately there is a liability to the recurrence of these attacks more or less frequently repeated, and each succeeding attack leaves the mucous membrane more and more injured, until the sinuses become lined with the polypoid mucous membrane and sometimes even contain polypi, accompanying which is the constant purulent secretion of the chronic condition.

That a chronic discharge of pus from these sinuses frequently takes place without presenting the definite symptoms of empyema is certain. Where the nasal passages are free and the openings from the sinuses are large the swollen mucous membrane may not be able to completely retain the discharge, and for some time at least the characteristic symptoms of empyema do not occur. Sooner or later, however, in many of these cases, the mucous membrane lining these cavities becomes more and more polypoid, or swells up much more during an attack of acute rhinitis; a complete stoppage of the opening occurs, and a true empyema results. Very often the nasal mucous membrane becomes polypoid and true polypi may result, possibly due to the irritation caused by the retained discharges, and possibly to the direct spread of the inflammatory process.

Of course, the question of the direct spread of the inflammatory process from the nose into the sinuses, and *vice versâ*, may arise. It has been asserted that this never occurs. Bosworth, *e.g.*, in his "Diseases of the Nose and Throat," Vol. I., p. 466, states that "a catarrhal inflammation of the nose is the result of local conditions, which do not in any degree probably operate in the maxillary sinus." "In other words, so-called nasal catarrh is a perversion of function of the normal respiratory apparatus of the nose, and its causes only operate on those tissues, and would have no effect on the delicate membrane lining the antrum." And still patients with acute nasal catarrh constantly exhibit undoubted symptoms of acute catarrh of their sinuses.

No doubt a chronic condition might arise from the blocking in and decomposition of the normal discharge within these cavities by some condition of the nose itself, in which the nasal passages are small and the openings into the sinuses are blocked by polypi, polypoid mucous membrane, or an hypertrophied condition of the mucous membrane over the middle turbinate. If the normal discharge got blocked in and decomposed it would set up a certain amount of irritation in the lining membrane of these cavities, and the oftener the recurrence the more sure the development of an inflammation of the membrane itself. Then again the process may be initiated in the maxillary antrum by the formation of an abscess or a chronic inflammation round the fang of a decayed tooth, seeing that we often have the outer alveolus of the molar teeth in the floor of the antrum.

Foreign bodies in the sinuses and tumours, whether benign or malignant, may by their presence set up irritation, or, by blocking up the openings, cause a retention of the discharges and so produce suppuration.

In the case of the ethmoidal cells circumstances are somewhat different. Here there are a mass of cells separated more or less from one another and varying in size. When inflammation occurs in the lining membrane of these, this undergoes the usual changes resulting in a myxomatous condition. This, if it exists for some time, gives rise to a distension of the cells, and even in some cases to a protrusion of the myxomatous tissue from the cells, and the further development of these buds, as it were, into true polypi, which, usually of small size, are to be found more or less blocking up the infundibulum, the hiatus semilunaris, and the space between it and the wall of the maxillary antrum. Sometimes they are also to be found in the superior meatus, especially posteriorly. That this is the origin of only a small proportion of cases of nasal polypus has been amply shown by Zucherkandl, who found them to arise from the ethmoidal cells in only 9 per cent. of his cases. ("Zucherkandl: Normal und Pathologische Anatomie der Nasenhöhle," Vienna, 1882, p. 78, &c.)

As a result of this distension of the cells, these are found to gradually extend in the body of the ethmoid, and even into the middle turbinate. Where this occurs it gives rise to a distinct bulging of the middle turbinate into the middle meatus, pressing close against the septum and forming a distinct ovoid mass. In this way the middle tubinate, or, at any rate, a large portion of it, is converted, as it were, into a large bony cyst. At the same time, the nasal mucous membrane lining this frequently becomes myxomatous, and thus this condition may come to exist both inside and outside these distended cells. Absorption of the thin, bony walls of the cells also takes place, and in some cases the mucous membrane may be so thinned as to give the feeling of bare bone to a probe—especially as the thin membrane is easily ruptured by even gentle manipulation. This same feeling can sometimes be got in even a healthy nose.

While these changes have been occurring there is present the usual increased secretion which helps to the cellular distension, the normal openings of the cells being already partially if not wholly closed. Failing immediate resolution, or if there are closely repeated attacks, chronic suppuration results, the pus finding its way either through the anterior or posterior ethmoidal cells.

That suppuration in the antrum and other sinuses is not an infrequent accompaniment of the disease of the ethmoidal cells cannot be wondered it. In fact, many of the worst cases which are met with are thus complicated. The same bulging of the ethmoidal cells and development of polypoid and swollen mucous membrane in the hiatus semilunaris, where most of the sinuses have their openings, must lead to stoppage of the middle meatus and along with it the openings of these sinuses, leading to the retention of their normal secretion and possible subsequent decomposition, with the results which have already been indicated. However, in most cases, the same causes which give rise to the ethmoidal trouble would also give rise to that in the other sinuses; although, in the case of the ethmoid, isolated cells would be more apt to get their openings blocked, and then the disease would probably have laid a hold on them sooner.

To sum up the connection between nasal polypi and suppuration in the accessory cavities: Myxomatous degeneration of the mucous membrane of the nose and polypi differ but little histologically. The formation of nasal polypi is favoured by the position in which they occur, and probably by slight differences which exist in the mucous membrane. They occur especially along the outer and lower edge of the middle turbinate and along the hiatus semilunaris, although in a non-pedunculated form they are by no means uncommon growing from the septum and inferior turbinate. The œdema and increase in size of the polypi must also be considerably aided by their pendulous position, and to a great extent by the suction action of the air as it passes through the nasal passages, especially when respiration is more or less through the middle meatus only. Their coexistence with suppuration in the sinuses is probably in most cases due to both being the result of a similar cause. Although, on the one hand, the blocking up of the normal openings into these cavities by polypi may lead to empyema, on the other hand the irritation caused by chronic suppuration in the nasal cavities may produce the polypi. The independence of the one from the other is established by the fact that we can have empyema without polypi and polypi without any trace of retained discharges in the cavities. That their origin is due to a chronic irritation of the nasal mucous membrane is proved by the fact that there is always more or less round-celled infiltration, and there are usually also present other signs of inflammation in the nasal mucous membrane. And, finally, suppuration in the cavities may fairly well be considered as due to a chronic inflammatory process, be it from chronic catarrh, the retention and decomposition of the nasal discharges, foreign bodies within the cavities, or abscess due to the irritation of the fangs of a carious tooth.

